# Switching Dynamics in an Interpersonal Competition Brings about “Deadlock” Synchronization of Players

**DOI:** 10.1371/journal.pone.0047911

**Published:** 2012-11-05

**Authors:** Akifumi Kijima, Koji Kadota, Keiko Yokoyama, Motoki Okumura, Hiroo Suzuki, R. C. Schmidt, Yuji Yamamoto

**Affiliations:** 1 Faculty of Education and Human Sciences, University of Yamanashi, Yamanashi, Japan; 2 Graduate School of Medicine, Osaka University, Osaka, Japan; 3 Division of Applied Physics, Faculty of Engineering, Hokkaido University, Hokkaido, Japan; 4 Japan Society for the Promotion of Science Research Fellow, Tokyo, Japan; 5 Faculty of Education, Shizuoka University, Shizuoka City, Japan; 6 Graduate School of Education and Human Development, Nagoya University, Furo-cho, Chikusa, Nagoya, Japan; 7 Psychology Department, College of the Holy Cross, Worcester, Massachusetts, United States of America; 8 Research Center of Health, Physical Fitness and Sports, Nagoya University, Furo-cho, Chikusa, Nagoya, Japan; Hungarian Academy of Sciences, Hungary

## Abstract

In competitive sport game behavior, certain interpersonal patterns of movement coordination evolve even though each individual player only intends to exert their own strategy to win. To investigate this interpersonal pattern formation process, we asked pairs of naïve participants to engage in a play-tag game in which they had to remove a tag fastened to their partner's hip. Relative phase analysis of the players' step towards-away velocities indicated that anti-phase synchronization evolved across 10 repetitions of the game. We clarified evolution of this synchronization process using a dynamical model with an attractor (at relative 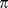 phase) and a repeller (at 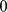 relative phase) and discuss the self-organized nature of model and its ability to embody general solution for martial art interpersonal coordination.

## Introduction

Athletic competitions inevitably end with a winner and a loser. If one could assign weight 1 for winner, −1 for loser, and 0 for both when the match resulted in draw, any sports game can be thought as zero-sum game that is, a game in which the sum of weights assigned to the players is zero (about this perspective, see Text S1 for further discussion). There are two types of competition in sport. The first kind is a sport event in which individual player can exert maximum ability of her or himself with least disturbance by opponents. Typical examples of these sorts of sports are track and field or swimming. For example, a track athletes' performance is interfered with by other athletes only in limited situations like when another runner takes an initiative position in long distance track race). The second kind of competition is a sport event in which, each player tries to maximize their performance by disturbing and minimizing opponent's performance. Typical examples of such sports are the martial arts contests (e.g., wrestling, fencing and judo), or one-on-one ballgames (e.g. tennis or badminton). Players in these games exert a strategy (i.e. attacking or defensive behavior) in which they try to earn points, and concurrently, not allow his or her opponent to gain points. Such one-on-one interpersonal sport events can be labeled as incomplete zero-sum games with finite strategies, as a player never knows completely his opponent's strategies that are being picked up from finite number of alternatives.

Previous study of sports games has revealed that experts tend to adopt a mini-max strategy: to minimize one's own possible maximum deficit, rather than to maximize the possible minimum benefit of one's self (the later is called maxi-min strategy). Such studies, however, only analyzed this strategy in partial situation of whole game, such as penalty kicks in soccer [1], [2], or those serving in tennis [3], [4]. There have not been any studies that investigate player's strategies across the entire duration of a game. Dynamical system analysis of tennis by Palut and Zanone [5] is one of the attempts to do so. They analyzed two players' movement coupling during cooperative or competitive rallies, and analyzed not only each player's action but also the phase relationship between two competitive players' movement. They found evidence for in-phase and anti-phase coupling of the direction of two players' movement.

However, because so much of the player movements were not in in-phase or anti-phase, the authors could not conclude that these two phases modes were inevitable or intrinsic states of movement coupling determined by structure of tennis. They speculated that the result was caused by weakness of visual coupling between the players. Although there is evidence for the strong effect of vision on interpersonal synchronization [6], another possibility is that player's movement (velocity) itself may not have been the best variable to describe game behavior in tennis. Certainly, velocity is basic and rudimentary variable: however, a player's velocity is not necessarily determined uniquely by the attacking or defensive strategies adopted.

Dynamical systems analysis has been used to model the coordination behavior in physical systems (e.g. clock pendulums [7]; metronomes [8]) and for biological multi agent systems (e.g. dynamical stability of multispecies' population in ecosystem [9]; extinction in ecosystem [10]). However, the essential variables in these analyses are clearly defined and capture the systems dynamics so that evolution of these systems can be modeled in terms of mathematically defined attractors in a phase space. When we attempt to apply this analysis to sports game, however, we need to extract the essential variables that represent evolution of the game dynamics that are already implicit in rules of the game.

Using this strategy Braun et al. [11], [12] have modeled multi-agent motor interactions using a dynamical systems analysis based on game structure. They devised an interpersonal reaching task in which there was a continuous change of resistance on each player's arm based upon the position of their partner's arm. A function that translates one's hand position to the resistance of their partner's hand was specified to correspond to payoff matrices defined across several cognitive games. The researchers found that pairs of players coordinated their movements to satisfy an equilibrium defined by both players' strategy that conformed mini-max solution for each, so that, attractors emerged in the interaction between the movements of the pair that embodied the optimal solution of the game. This result was found for non-cooperative games such as prisoner's dilemma with a unique Nash equilibrium [11], but it was also true for several cooperative games with multiple equilibria [12]. Thus, attractor dynamics of interpersonal perceptual-motor coordination can be determined by game structures defined by payoff matrices of the strategies adopted by players.

The tasks used by Braun and colleagues have a structure essential to that of one-on-one sport games (i.e., ball games such as tennis, or a martial arts contest such as judo), in which each player's behavior taken at a given time continuously constraints the opponent's next behavior. However, such constraints bring about a consequence in sports games that do not occur in the Braun task. One-on-one sport players' interaction recursively constrains their behaviors until the game is over, so that, the games become protracted when the players have some level of game experience and knowledge about game strategies. Such ‘deadlock’ situations are the essential nature of competitive sport games. Therefore, the goal of dynamical systems analysis of sport games could be defined as the exploration of attractors dynamics that correspond to the evolution of deadlock equilibrium solutions through the course of the interaction as constrained by the strategies (i.e. payoff) adopted by the players.

It is difficult, however, to determine exclusive one-on-one correspondence between movement patterns and game strategies because in real sports games the correspondence between movement patterns and strategies is very rich. Consequently, in the current study, naïve participants will play a prototype of competitive sport game, that we call play-tag, which contains strategies that are inherent in martial art competitions. In play-tag, pairs of participants try to remove a tag fastened to their opponent's body so that each movement they make can be defined as one of two strategies: approaching to one's opponent by stepping close, or avoiding one's opponent by stepping away. Therefore, each step a player makes is uniquely defined by these two game strategies. Our method is to express the time sequence of the switching process between these strategies as defined by a signed continuous variable that indicates velocity to step close as negative (arbitrarily) and the velocity to step away as positive. We believe that the relationship of these two time series will reveal a stable phase coupling across game repetitions that will represent a deadlock solution that is intrinsic to dynamics of play-tag; and moreover, this phase coupling represents a general solution that is determined by the payoff structure of the play-tag rules. Thus, the aim of the current research is to investigate the interpersonal behavioral attractor layout that evolves as a function of habituation or learning of the tag players. Any evolution of the attractor layout must be due to individual switching patterns of not-to-lose or to-win strategies through the course of the trials and not due to evolution of game structure itself that is static and defined on the basis of game rules. This function is similar to social coordination systems of biological crowd behavior (flocks of birds [13]–[15], schools of fish [16], [17]). In these collective behaviors, the geometrical or temporal coordination patterns observed were organized by the individual element's behavior. By investigating the coordination pattern between competitive tag players' movement, we are attempting to provide some insight into the general model of embodied attractor layouts that are intrinsic to optimal solutions found in one-on-one sport contest such as those found in the martial arts.

## Materials and Methods

### Participants and pair assignment

Ten male participants were recruited from Fukuyama-Heisei University soccer team. All of them provided written informed consent prior to the experiment and were included in the study. Procedures were approved by the Internal review Board at the Research Center of Health, Fitness, and Sport at Nagoya University and conformed to the principles expressed in the Declaration of Helsinki. Mean (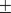 S.D.) age of participants was 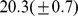 yrs. Mean height and weight of eight of the participants were 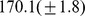 cm and 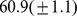 kg, respectively. Heights of remaining both two participants were 180.0 cm, and had weights of 76.0 kg and 78.0 kg, respectively. Participants with similar body size were assigned to the same pairs as presented in Table S1.

### Task: Play-tag

The participants were instructed to remove one of two tags their partner had fastened to their hips with Velcro tape. The tags (those used for flag football; Evernew Inc., JPN) were 2.5 cm×90.0 cm nylon cloth with 45.0 g weights. Participants were also told not to step out from 5.0 m×5.0 m square marked with tape on a boarded floor. Before a game was started, the participants in each pair faced one another. The trial began when the experimenter said ‘go’. Participants were further instructed not to cover their tags with their hands to prevent partner from removing it. A trial was completed when either participant had taken one of his partner's two tags. Participants performed 10 trials of the game with same partner, and tried to obtain their partner's tag as many times as possible. Descriptive numerical information about the participant pairs is presented in Table S2 (also see Video S1 showing an example of participants' movement in play-tag).

### Measurement procedure

The five pairs performed the experiment across two days (three pairs on one day and two pairs on the other). The rules of the play-tag game were explained about 30 minutes before the first trial began. While participants listened to instructions, the experimenter attached reflective marker to the top of their head, and fastened the tags to their hips with Velcro tape. A heart rate sensor (Pro Trainer 5, Polar Inc.) was also attached to each participant's chest and data logger to their wrists to measure their degree of physiological fatigue during ten play-tag trials. An optical motion capture system with four cameras (100 Hz, OQUS, Qualysis Inc.) was used to record the two participants' movement trajectories during trials. After each trial, the participant pair was moved to an interview room where the experimenter asked them about their experience during the trial (for detail, see Text S2). Their answers were recorded using a video camera (DCR-DVD505, Sony Inc.). The pair was then allowed to rest in the separate room while another participant pair performed the task. All pairs completed 10 repetitions of this game-interview-rest session. Participants never saw the performance of another pair.

### Dependent measures

We calculated distance between participants in a pair to be able to detect the segments in which the distance was small enough not to be caught but to catch a tag fastened to opponents body. Phase coupling was measured using a relative phase analysis performed on the participants step toward-away velocities. We furthermore calculated the instantaneous product the participants step toward-away velocities to evaluate the switching process of of the step toward-away coupling (i.e., predator-prey role) that would be emerge during the course of each play-tag trial.

#### Distance and length of coordination segment

Participants' position trajectories were expressed as a time dependent vectors 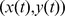 and 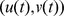. These time series vectors were calculated using software (Qualysis Track Manager, Qualysis Inc.), and smoothed using fourth ordered Butterworth filter with a cutoff frequency of 6 Hz. The time series of the Euclidean distance (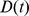) between two participants was calculated using the following equation:
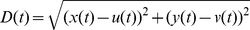
(1)


Time series of 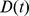 were used to identify the time boundary between game segments. Linear regression analysis was performed on the data using a moving time window (width = 0.3 s, numbers of data = 30). We determined the boundary between preparation and coordination segments, as the first point at which sign of regression coefficient turned from negative to positive (i.e., approximately as where the first point of regression coefficient reached to zero; see Text S3 and [18] for details on using linear regression analysis for segmentation).

#### Step toward-away velocity

**Figure 1:**
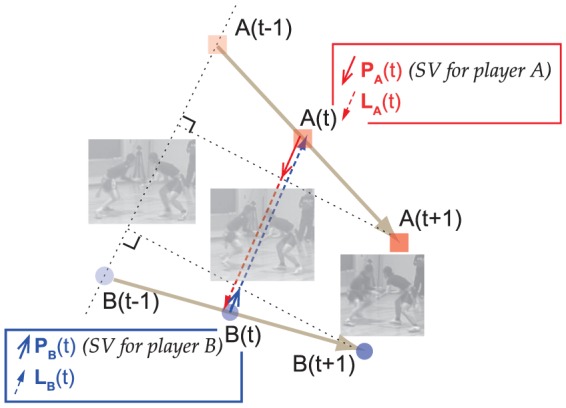
Schematic representation of SV(t). In this example, Participant A moves from position A(t−1) to A(t+1), during time from t−1 to t+1. SV(t) for participant A, depicted as 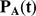 (red solid arrow), was defined as projection of vector 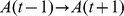 to vector 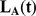 (red broken arrow). Sign of SV(t) was assigned negative if SV(t) directed toward opponent, and positive if SV(t) directed away from opponent. SV(t) for participant B also calculated using the same procedure (blue solid arrow). Note; Red and blue solid arrows denotes magnitude and direction of SV(t) for each participant at A(t) and B(t) respectively.

We also calculated the step toward-away velocity (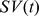) to estimate the movement of each participant (namely, A and B in the following text). To calculate these variables, displacement vector from time 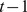 to 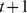 (as sampling frequency was 100 Hz, time lag in real time was 0.02 s) was defined as 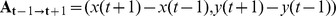 for participant A, and 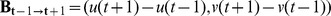 for participant B. Projection of vector 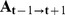 (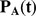) to a linked vector (for example in Figure 1, 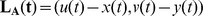) with direction from position of A to B at time 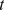, and that for B (Projection of vector 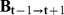: 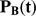; linked vector: 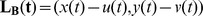 were also obtained by equation (2) and (3), respectively (also see, Figure 1).
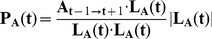
(2)

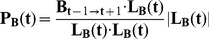
(3)


We then defined length of projection 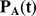 and 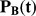, as SV(t) for participants A and B, respectively. Those values represent each participant's velocity in stepping toward to their opponent (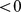) or in stepping away from him (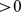).

#### Instantaneous frequency and phase

Instantaneous frequency 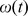 and instantaneous phase 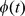 of the SV(t) fluctuation for each participant as well as the lag between both participants' frequencies (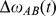) and phases (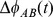) were calculated. To calculate these variables, we decomposed each participant's SV(t) time series to real and imaginary part using Hilbert transform formulated as equation (4),
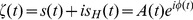
(4)where 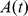 denotes amplitude and 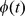, instantaneous phase. 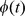 for each participant A and B, 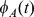 and 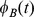 were obtained by calculating the arctangent of ratio of real part (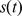) to imaginary part (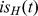) of the Hilbert transform formulated as equation (5) and (6) (also see Text S4 for details about the effectiveness of the Hilbert transform on the current SV(t) data),
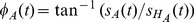
(5)

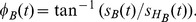
(6)


Because 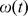 is the first order time-derivative of 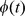 [19], [20], we obtained 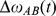 by subtracting 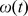 for participant B (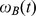) from those for participant A (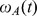). Finally, 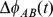 was obtained by equation (7) [21].
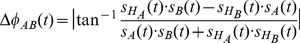
(7)


#### State transition probabilities about step toward-away (predator-prey) relationship

Relative phase 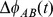 indicates the relationship between two players' movement. 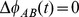 denotes that both participants simultaneously step toward or away to each other, on the other hand 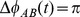 denotes that one step toward and other step away to his opponent and vise versa. We furthermore divide 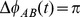 state into two states of coordination, a state in which one participant A steps toward and B steps away and a state in which A steps away and B steps toward.

In order to confirm a transitory sequence between these two states, we first calculated instantaneous product of two participants' SV(t) (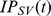) for entire duration of each trial using equation (8).
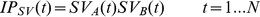
(8)whereas 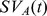 denotes 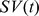 for participant A and 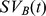 denotes that for participant B (i.e., the signed length of 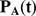 and 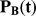 in equation (2) and (3), respectively). N denotes number of data ( = trial duration (s)×sampling frequency (100 Hz)) . Therefore 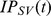 is the product of both participants 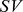 at a time point 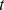 and the sign of 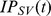 denotes patterns of two participants movement coordination at 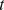. If 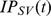 is positive, the two participants step simultaneously in the same direction with respect to their opponent (i.e., toward or away) at 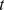. On the other hand if it is negative, the participants step in the opposite directions with respect to their opponent (i.e., one steps toward and other steps away or vice versa).

We then calculated zero-crossing point of 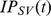 at which the value turned from positive to negative (i.e. where the signs for each participant's 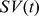 changed), and confirmed which participant stepped toward (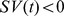) and which stepped away (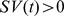) after each zero crossing point. Participant who step toward can be seen as predator, and others who step away can be seen as the prey. Thus each participant who moved in anti-phase manner (i.e. 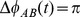) must have either a predator or prey role as denoted by the sign of his 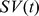. The role assigned at the time 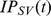 turned from positive to negative remains until the sign changes again. We then, obtained event sequence of predator-prey role alternation and calculated probabilities of these switching of roles or transitions of state.

In play-tag situation, if A stepped toward at a given time then either of A or B can step toward the next time, and also, if B step toward at a given time likewise either A or B can step toward at the next time. We therefore calculated each probability of 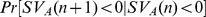, 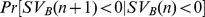, 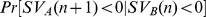, 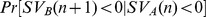, whereas 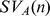 denotes 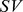 for participant A at 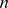
th zero-crossing point. These variables, especially 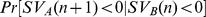 and 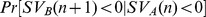 indicate the activity of role switching behavior. (See Figure S1 for more detail on the detection of these switching events)

## Results

### Length of coordination segment

**Figure 2:**
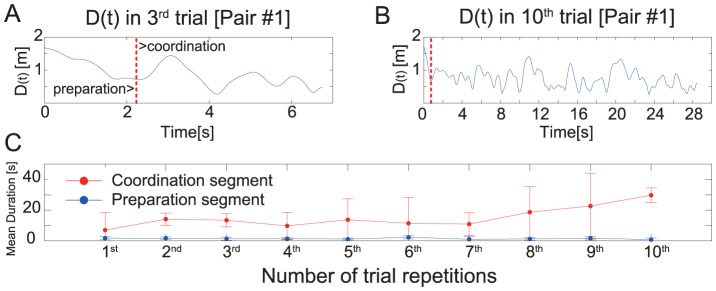
Examples of the time series of between-participant distance D(t) and mean duration of each segment. Examples of the time series of between-participant distance D(t) observed in the shortest (A) and the longest trial (B) of pair #1. Vertical dotted line in each panel indicates a time boundary of segment translation (preparation to coordination). Mean duration of each segment observed in each trial was shown in panel C.

The length of coordination segment in each of the 10 trials was calculated, and differences of segment duration between trials were tested using one-way repeated measures ANOVA. An effect of trial repetition on segment duration was statistically significant (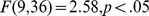). The trend presented in Figure 2C indicates that the duration of coordination segments was lengthen by repetition. Indeed, lengths of trial #10 were significantly longer than trial #2, #3 and #7 according to Tukey HSD post hoc tests. An additional analysis of same design was applied to investigate the effect of repetition on length of preparation segment. This ANOVA did not reveal a significant effect (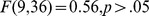; Figure 2C).

### Heart rates (HR) and subjective fatigue in the early-shortest and the late-longest trial

Next we focused on the trials with the shortest and longest durations in each pair to further investigate the extension of trial duration with trial repetition as indicated in Figure 2. Indeed the shortest trial for pairs #1 and #2 was the 3rd trial while for pairs #3, #4 and for #5 it was the 1st trial. By comparison, the longest trial, in pair #1, #3 and #4 was the final trial, while for pair #2 it was the 9th trial and for pair #5 it was 8th trial. Mean duration of shortest and longest trials is presented in Table S2. We investigated the effects of repetition or duration extension on physical fatigue of participants, by comparing heart rate of the participants in the early trials with shortest durations (early-shortest trials) to the later trials with the longest durations (late-longest trials) (Table S3). There was significant difference between peak heart rate of early-shortest and late-longest trials (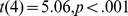). We also found a significant difference (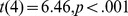) for the peak HR reserve (calculated using Karvonen's formula; [22]). These results indicate that the intensity of early-shortest trial was moderate whereas the intensity of late-longest trial were relatively greater for the participants. On the other hand, for the heart rate at trial onset, a t-test did not reveal a significant difference between early-shortest and late-longest trials, (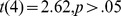). These results suggest that there was no accumulation of fatigue during repetition from the early shortest to the late-longest trials, although the exercise intensity itself was significantly higher in late-longest trials. In support of this result, none of the participants reported any subjective cardio-respiratory or muscular fatigue during 10 repetitions of trials. Exercise intensity level estimated using heart rate reserve according to the ACSM guideline [23] was actually at a low level at early-shortest trials, and reached only a moderate level in late-longest trials.

### Lag between two participants' instantaneous frequencies (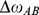)

**Figure 3:**
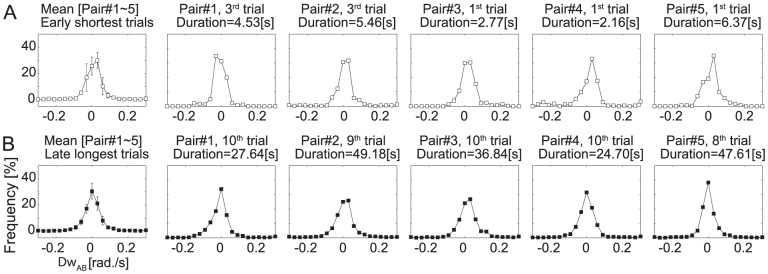
Distribution of lag of participants' instantaneous frequency (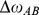). A: Early-shortest trials for each pair. B: Late-longest trials for each pair.

The distribution of the frequency lag observed in the early-shortest and the late-longest trials in each pair is presented in Figure 3A and B (total N = duration(s)×100 Hz). The lags observed in every pair are distributed around zero, and this fact was not altered by trial repetitions in any of the 5 pairs. These results indicate that the two participants of a pair consistently moved at similar frequency from the early-shortest trials to the late-longest trials. To test the statistical significance of distribution bias due to trial repetitions, distributions of frequency lags across 21 regions (from -0.5 to 0.5 rad/s) observed in each of early-shortest and late-longest trials were submitted to a 21 (lag region)×2 (repetitions: early-shortest and late-longest) repeated-measures ANOVA. A main effect of repetition was not significant, although there was significant main effect of lag region (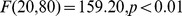). Post hoc analysis (Tukey HSD) revealed frequencies in 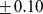 rad/s region were greater than those in any other region, and moreover, frequencies in 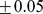 rad/s were greatest among them (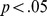). The nonsignificant effect of repetition indicates that Gauss shaped distribution with single peak at 0 was not altered such that the participants in a pair moved at a similar frequency throughout the course of the 10 trials. The significant effect of lag region indicates that each participant had to follow their opponent to avoid or engage in their attack so that, they had necessarily to move in same rhythm.

### Lag between two participants' instantaneous phases (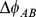)

**Figure 4:**
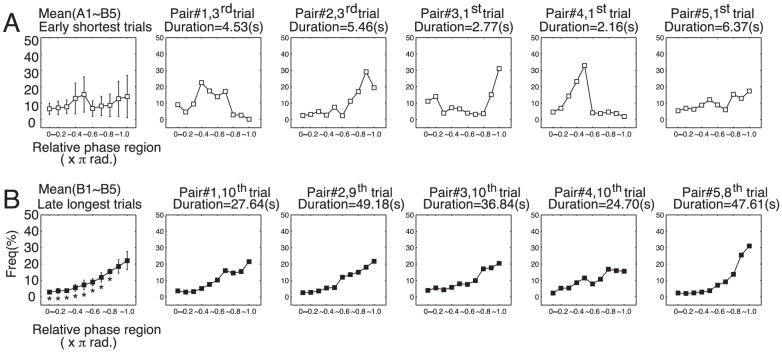
Distribution of time frequency (in %) of lag between two participants' instantaneous phase (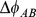). A: Frequencies observed in early-shortest trials. Mean (blank squares) and SD (error bar) for 5 pairs (left panel) and frequencies observed in each pair (right 5 panels). B: Frequencies observed in late-longest trials. Each ticks in abscissas indicates relative phase region that divide 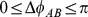 into 10 regions (each range: 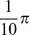). Asterisks denotes the regions in which frequencies were significantly lower (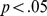) than those for 
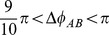
.

We calculated the distributions of phase lag across 10 regions (from 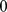 to 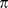; 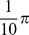 for each region), for the early-shortest trials (Figure 4A) and late-longest trials (Figure 4B). To confirm attractor layout in relative phase space, we submitted the distributions to a **10** (relative phase region)
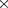
2 (repetition: early-shortest and late-longest) repeated measures ANOVA. The ANOVA yielded significant main effect for lag region (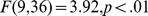) and also yielded significant interaction between lag region and repetition (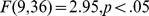). A significant simple main effect of region was not observed for early-shortest trials (Figure 4A left panel; 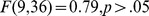), but was observed for late-longest trials (Figure 4B left panel; 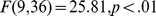). Post hoc analysis (Tukey HSD) indicated that phase lag was more concentrated in both anti-phase regions. Mean frequency observed in 
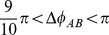
 region were significantly greater than frequencies for regions that include 
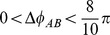
. (See asterisks denoted in Figure 4B left panel).

The results shown in Figure 4A suggest that the participants in early-shortest trials would move in unstable manner with less coupling consistency than in the late-longest trials. A distribution with a peak at 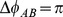 suggests that participants were moving in an anti-phase coupling, in which they might step close to the opponent while their opponent stepped away or they might step away while their opponent stepped closer. In contrast, a distribution with a peak at 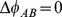 would suggest that participants were moving in an in-phase manner in which they might step close to opponent as opponent also close to him, and step away as opponent step away. The less differentiation of such in-phase and anti-phase modes in the early-shortest trials indicate that, there was no systematic phase-locked coupling pattern in these trials. This kind of coordination tends to be unstable in which the equilibrium of participants' risk for losing tends to break. This situation in early repetitions, therefore led to either participant's “sudden death” in a relatively short duration.

This unstable situation, however, appeared less in the later trials as indicated by the extension of trial duration. Different shapes of distribution between 5 pairs all converged to a common shape with a peaks at 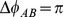, and indicates that participants synchronized in an anti-phase manner (presented in right five panels aligned in Figure 4B). Given the drastic extension of competition time at about the 8th to 10th trials (see Figure 2C), a so-called “deadlock” mode could have been brought about by this anti-phase synchronization.

**Figure 5:**
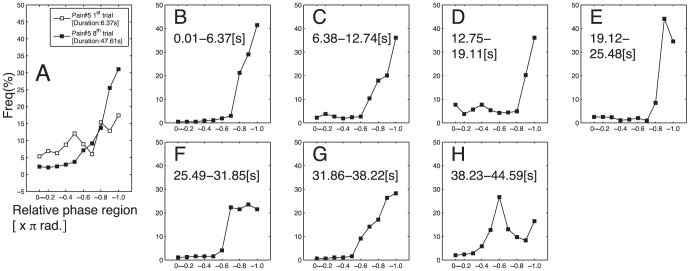
Distribution of instantaneous relative phase (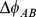) measured for the early-shortest and late-longest trials of a typical pair (#5). A: Distributions calculated for all durations of early-shortest trials (blank square) and late-longest trials (filled square). B–H: Distributions calculated for each 6.37 s time series segments of a late-longest trial.

Figure 5 indicates that the peaks seen at 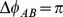 are not simply due to an increase in the amount of data. In this figure, the distribution of phase lag for an early-shortest and a late-longest trial of a typical pair are plotted in panel A (each was indicated by open squares and filled squares, respectively). Filled squares in each of panel B-H indicate the distributions of phase lag for the portions of the late-longest trial that were of the length of early-shortest trial. It can be seen that the peak around 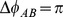 are present throughout the trial. These results give an insight into the participants' coordination in the late-longest trials that is an anti-phase manner (
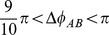
) dominated up to or more than 50% of any period duration during these trials. This anti-phase domination was confirmed again in relative phase analysis performed to portions shorter than 6s (results were not shown).

**Figure 6:**
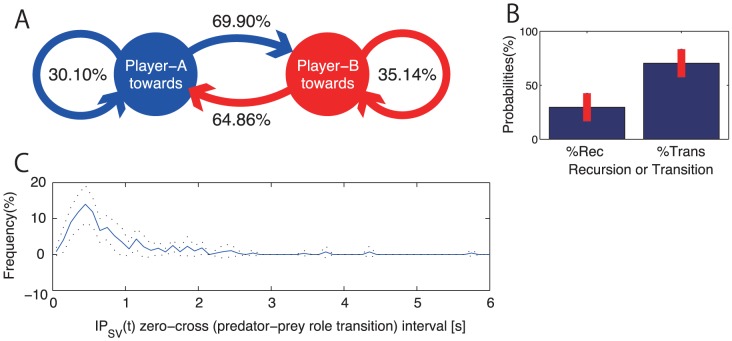
State (predator-prey role) transition probability and time interval of transition in anti-phase dominated late-longest trial A: State transition probability diagram of role alternation sequence observed in late-longest trials performed by pair #1 to pair #5. Diagrams for individual pair were shown in Figure S2. B: Mean of 5 pairs' transition probability (%Trans: mean of 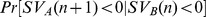 and 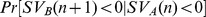 of individual pair) and recursive probability (%Rec: mean of 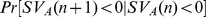 and 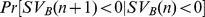). Error bar indicates SD. Difference between %Trans and %Rec was statistically significant (p<.05). C: Mean frequency (in %) of time interval for state and recursive transitions and recursion. Solid line indicates mean of 5 pairs and black dotted line indicates 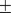 SD.

In Figure 6A, we display transition probabilities between two states of anti-phase coordination in late-longest trials. The diagram indicates that probability for state transition (i.e., a predator who stepped toward turned from A to B or B to A between successive zero-crossing point of 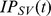) was greater than those for recursive transition, also called self transitions, that start and end in the same state (i.e., predator remained A or B between successive zero-crossing point of 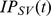). Probabilities in Figure 6A were calculated based on the data concatenated late-longest trials performed by pair #1
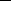
#5. We statistically compared probability for state transition to those for state recursion, by calculating the mean of two transition probabilities and those of recursive transitions for individual pair (a diagram for each pair is presented in Figure S2). We submitted these probabilities to one way (2: state and recursive) repeated measure ANOVA. The results indicates that the probability for state transition was significantly higher than those for recursive transition (F(1,4) = 12.28, p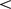.05, Figure 6B). Thus anti-phase coordination that dominated in late-longest trials was formed by the role transition between the two participants. Additional analyses of the time intervals between 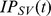 zero-crossing points were performed to confirm the time order of the role alternation. According to Figure 6C, the interval seldom exceeds 2.0 s and concentrated to 0.4–0.5 s region (median = 0.630 s). Thus the role transition would be spontaneously evolved with a 70% probability in the two players' role transition-recursion sequence that altered most in 1.0 s intervals.

**Figure 7:**
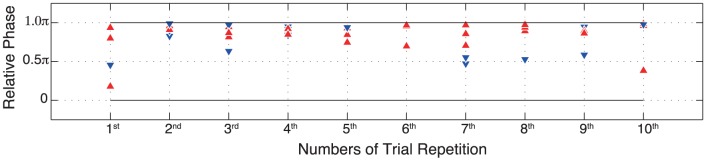
Phase lag (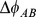) and a role of winner at which either player's tag was taken in the five pairs. Red triangle markers denote winner stepped toward (i.e. predator role) and blue triangle denotes winner stepped away (i.e. prey role).

We also analyzed the phase lag at which a player's tag was taken. Figure 7 indicates the lag for each trial performed by the five pairs. The timing was defined as the point at which the hand that held tag begins to move backward. The effect of repetition on this phase lag was not statistically significant, and the plot shows that the player took opponent's tag at 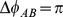 in most cases. We attempted to confirm whether the winner stepped toward or away when he took his opponent's tag using 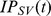 (as denoted by color and shape of markers on Figure 7), however, no trends can be clearly observed.

## Discussion

### Self-organized Attractor layout evolved in game repetition

While both participants' frequencies were similar from the onset of the trials, strong phase locked synchronization only appeared in the relatively later trials (8th to 10th trials). In early-shortest trials, there was less phase synchronization, even though both participants' frequencies were similar. Through accumulation of game experience participants became synchronized in anti-phase manner. The two participants were coupled in later trials, where as in the early trials, they only oscillated more independently which often resulted in sudden death. Throughout the attractor layout evolution, our participants intended only to catch opponent's tag (and not to have their own tag caught), and undoubtedly, their goal was not explicitly to synchronize in anti-phase. This anti-phase synchronization evolved through the competition by our participants who gradually learned the strategies that comply with the game demands.

In several biological crowd behaviors such as flocks of birds or schools of fish, functions similar to tag players' synchronization have been observed. For example tuna (migrant fish that can swim great distances per year) school in a diamond shape in order to minimize each individual fish's energy consumption [16]. Additionally, trout reduce their muscle activity [17] or oxygen consumption [24] by exploiting vortices produced by obstacles that might include neighboring fish in a school. Individual fish clearly do not intend to minimize each other's energy consumption while swimming long distances or in turbulent water. Thus, fish schools are self-organized: Each fish only adopts a strategy to minimize their own energy consumption and does not intend to form well-ordered school geometry, however, this geometry contributes to maintain efficiency for each of the individuals.

The well-ordered anti-phase synchronization in play-tag behavior also seems be self-organized. Different from dancing behavior, the players clearly did not intend to synchronize with each other, but are competing to take their opponent's tag without their tag being taken by their opponent. In fact the tag players' interaction is different from that of a fish school in that the players are competing with one another. Both players learned to switch their strategy during the course of game play, and the evolved dynamics which caused the players to remain in a stable competitive state for a long time (as shown in Figure 2C), in spite of their intention to win the game as quickly as possible. In addition, this deadlock (anti-phase) synchronization does not seen to have evolved not under an energetic constraint in that the players were not exhausted at the onset or even during late-longest trial (see Table-S2). Instead, the factors that constrained the emergent anti-phase synchronization can be defined in terms of the social interaction specified by game's structure or rules.

### Nature of tag-players' synchronization

The characteristics of synchronized oscillators have been repeatedly observed in interpersonal coordination tasks in controlled experimental treatments [6], [25]. These characteristics have been hypothesized to be universal properties of rhythmic coordination, in that they have also been seen in intrapersonal interlimb coordination [26], [27], as well physical systems such as metronomes [8] or Huygen's clock pendulums [7]. In both the cases of intrapersonal or physical systems, the direction and amount of phase lag can be controlled by changing the difference between the oscillators' inherent frequencies. Several studies on interpersonal coordination also have reported that there is linear relationship between difference between the oscillators' inherent frequencies and an observed phase lag (for review, Schmidt, Fitzpatrick, Caron, & Mergeche [28]). In that Nessler and Gilliland [29] reported that synchronization between treadmill walkers was influenced by difference of walkers' body length, difference in the mass of the players in pair also might alter tag players' coordination pattern. This possibility can not be investigated using the current play tag data because the body lengths of the participants in a pair were physically matched. One can speculate that the synchronization between players shown in Figure 3 and Figure 4 might be have been enhanced by this matching treatment; however, to fully test this hypothesis, we would have to confirm that synchronization patterns do vary with differences in inertia of the two players' bodies for example, by differentially weighting the legs of the participants.

Recent research reported that such human synchronization can be enhanced by psychological treatments as well as physically altered frequency (for review, Riley, Richardson, Shockley, & Ramenzoni [30]). For example, interpersonal synchronization in cooperative task that needed spatial accuracy could be enhanced by the task demand of accuracy, or by the increasing the difficulty of stabilizing posture [31]. Other research has found that two musicians both with high prediction performance exhibit more accurate and stable tapping synchronization of higher accuracy and lower variability than dyads both with low prediction performance [32]. Other research has found that three expert football players maintain synchronization during 3 vs 1 ball possession task under task constraints in which the three attackers tried to keep possession from a defender and pass the ball to other attackers as much as possible within a 6-m square [33]. These effects of non-physical constraints, such as attentional demand to task or partners' movements, suggest that the synchronization observed in current data could be a product of each participant's increased attention or prediction to their opponent's movement based on understanding of game structure (see participants' verbal report below). In addition, Oullier, de Guzman, Jantzen, Lagarde, & Kelso [34] revealed that prior experience with movement synchronization through visual information exchange persisted even after visual information was removed. They call this persistence “social memory”, and claimed the phenomenon may be useful in studying interpersonal behaviors in general, including sport or game behaviors. Although their term “social” is different from ours, the clarity of the concept of coordination pattern persistence or carryover might be benefited by further investigation of individual player's learning to synchronize with opponent in play tag.

### Game structure of play-tag that constrained anti-phase deadlock coordination

In an analysis of interpersonal coordination in a real sport game, Palut & Zanone [5] claimed that tennis players coordinated their movement in in-phase (both move to same direction), in anti-phase (each moves to opposite direction) or in some games, the players moved in more complex mode when the number of rallies increased, where two or more relative phases switched alternately. Different from tennis, few phase transitions between in-phase, anti-phase or other relative phase were observed in current play-tag data. This difference is likely due to difference between game structure of tennis and play-tag. In tennis, a player's movement must follow the course of opponent's shot and consequently one player can lead opponent's movement by the course of shot itself. Under this constraint of the game, tennis players attempt to hit the ball to a place that their opponent can hardly reach. In order to achieve this goal, players sometimes have to break an ongoing pattern of phase coupling (e.g. in-phase or anti-phase) to avoid an opponent's attack. Players then would be forced to actively change a pattern of phase coupling. But in play-tag, on the other hand, participant can move anywhere in 5 m×5 m court without any risk of losing the game (that is relatively unconstrained compared to tennis), even though the locations of target tags are fixed to opponents' hips (relatively constrained compared to tennis). In play-tag, our participants strategy is to maintain a distance to catch an opponent's tag but also to not have his own tag get taken (See, Figure 2A, B). At this distance, they can reach their opponent's tag but at the risk of having their own tag taken. In this situation, when one reaches their hand to the tag, he, a “predator”, but might turn to “prey” at the next moment. Therefore the roles of each participant are actively altered during a trial; the anti-phase coordination itself is not altered as represented in Figure 5, but the role alternatively switched as represented in Figure 6. This constraint might be the essence of the play-tag game structure. Consequently, participants who had experienced several trials of the game found it necessary to move in an anti-phase manner and this might then have brought about the “deadlock” synchronization that occurred in the later play-tag trials.

### Play-tag game strategy embodied in anti-phase synchronization

As explained above, the two players have to trade-off purposes to obtain an opponent's tag without losing their own. An anti-phase coupling would contribute to a decreased risk for both players to lose in of a game with such a structure. In a study by Braun et al [11] two players regulated their energetic cost during reaching movements according to payoff matrix that was configured apriori. Although the players could not know completely about the payoff, each chose a strategy in which both have nothing to gain by changing only his own strategy. This solution is called Nash equilibrium, the typical solution to a non-cooperative game. Their findings indicates that this principle of an optimal solution in cognitive games could also be adopted in the instances of perceptual motor coordination. Attractors observed in current data would also function as an optimal solution of an apriori determined payoff. Play-tag is an artificial game that maintains one-on-one correspondence between two steps (step close and step away) and two strategies (approach and avoid). The player had to adopt an approach strategy while taking a risk to lose his tag, so he had to avoid while taking a chance periodically to take his opponent's tag. The optimal solution, therefore, inevitably became to predator-prey alternation, as embodied by anti-phase synchronization of steps.

Participant reports give some insight into each player's intention while playing the game. Most players after the early trials (until 2nd trial) reported about importance of timings or distance in reaching to the opponent's tag. Then about at 6th or 7th trial, the players became aware that the action to take their opponent's tags might turn to opportunity for opponent to take the player's own tag and finally at about 8th to 9th trial, they understood that the best chance to take opponent's tag would be at the moment which he lost his posture while trying to make a large step in to reach for a tag. Their adoption of a “not to lose strategy” provides the anti-phase step synchronization that conforms to a mini-max solution. According to Figure 7, however, the winner did not always step toward towards the loser. Indeed, as players reported for the later trials, sometimes they stepped away from the loser. Validity of players report about the function of step toward-step away behavior can be investigated by refining the criteria and measurement needed to identify when specifically a tag was taken.

In summary, our data indicates two novel findings. First, we have provided evidence for the existence of self-organizing processes of interpersonal coordination in a competitive sport game. In early trials, less strong phase-locking between players' steps were observed. In the later trials, the player's steps became synchronized in an anti-phase manner, so that the players' oscillations were putatively governed by an attractor layout with an attractor around 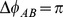 and a repeller around 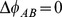. This synchronization could be a product of learning through which each player learned to anticipate opponent's approach and avoid movements. Second, the converged pattern of relative phase reflects the game structure of play-tag. The essence of the game is something like a tit-for-tat alternation of roles of two players in which actively alternated their predator-prey role. This switching dynamics that seems to bring about deadlock situation that is likely the result of a mini-max solution adopted by each player during the play-tag game. Finally, because dynamical systems and game theory constraints are thought general in real life competitive sports, or at least in two person games with body contact such as in martial arts contests, the synchronization pattern hypothesized in current study may be seen as a prototype for these games and future research can aim to further understand the dynamics of game structure and its relationship to expertise by confirming the use of attractor layouts like those we have proposed.

## Supporting Information

Figure S1Detail of role detection procedure. A: An example of time series of 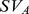 and 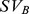 observed in a late-longest trial performed by pair # 5. B: 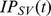 calculated from the data presented in Figure S1 A. Vertical dotted line indicates timings of role transition (or recursion) indicated by zero-crossing point of 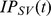.C: 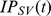 and it's zero-crossing points in 15–20s region in Figure S1 B. These intervals indicate from the time at which both participants' role changed until the time at which their role changed again. Note that each interval includes the phase in which both players simultaneously step toward or away (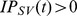) following to the phase in which one of them steps toward and the other steps way (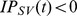).(EPS)

Figure S2 State transition diagram and role transition intervals for late-longest trial performed by the 5 pairs. Upper: State transition diagram for late-longest trial by individual pair. Lower: Frequency (in %) of time interval for role transition observed in late-longest trial performed by individual pair.
(EPS)

Table S1 Details of participants.
(EPS)

Table S2 Descriptive numerical information regarding each of #1 to #5 pair's performance. N. of Trials: Number of trial repetition. Dur.: Duration of a trial (in seconds). N. of TP.: Numbers of thrusts (and parries) in each trial. Thrusts were estimated from numbers of peaks observed in a time series of inter-participant distance (examples of data were shown in Figure 2A). Parries were estimated from numbers of valley. Win.: Winner in each trial.
(EPS)

Table S3 Heart rates and HRR before and during each trial.
(EPS)

Text S1 Comment about zero sum nature of the sport game.
(PDF)

Text S2 Comment about the potential effect of inter-game interview on game behavior.
(PDF)

Text S3Segmentation procedure according to two players' distance (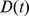).(PDF)

Text S4Effectiveness of Hilbert transform on current 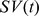 data.(PDF)

Video S1 Movie showing an example of play-tag.
(MOV)

## References

[1] Chiappori PA, S Levitt TG (2002) Testing mixed-strategy equilibria when players are heterogeneous: the case of penalty kicks in soccer. American Economic Review 92: 1138–1151.

[2] Palacios-Huerta I, Volij O (2008) Experientia docet: professionals play minimax in laboratory experiments. Econometrica 76: 71–115.

[3] Walker M, Wooders J (2001) Minimax play at wimbledon. The American Economic Review 91: 1521–1538.

[4] Hsu SH, Huang CY, Tang CT (2007) Minimax play at wimbledon: comment. American Economic Review 97: 517–523.

[5] Palut Y, Zanone PG (2005) A dynamical analysis of tennis: concepts and data. Journal of Sports Sciences 23: 1021–1032. 16194979 10.1080/02640410400021682

[6] Richardson MJ, Marsh KL, Isenhower RW, Goodman JR, Schmidt R (2007) Rocking together: dynamics of intentional and unintentional interpersonal coordination. Human Movement Science 26: 867–891. 17765345 10.1016/j.humov.2007.07.002

[7] Bennett M, Schatz MF, Rockwood H, Wiesenfeld K (2002) Huygens' clock. Proceedings: Mathematical, Physical and Engineering Sciences 458: 563–579.

[8] Pantaleone J (2002) Synchronization of metronomes. American Journal of Physics 70: 1–9.

[9] Afraimovich V, Tristan I, Huerta R, Rabinovich MI (2008) Winnerless competition principle and prediction of the transient dynamics in a lotka-volterra model. Chaos 18: 043103. 19123613 10.1063/1.2991108

[10] Coppex F, Droz M, Lipowski A (2004) Extinction dynamics of lotka-volterra ecosystems on evolving networks. Phys Rev E 69: 020902-1-4. 10.1103/PhysRevE.69.061901 15244611

[11] Braun DA, Ortega PA, Wolpert DM (2009) Nash equilibria in multi-agent motor interactions. PLoS Computational Biology 5: e1000468-1-8. 19680426 10.1371/journal.pcbi.1000468 PMC2714462

[12] Braun DA, Ortega PA, Wolpert DM (2011) Motor coordination: when two have to act as one. Experimental Brain Research 211: 631–641. 21455618 10.1007/s00221-011-2642-y PMC3102209

[13] Reynolds CW (1987) Flocks, herds, and schools: a distributed behavior model. Computer Graphics 21: 25–34.

[14] Hemelrijk CK, Hildenbrandt H (2011) Some causes of the variable shape of ocks of birds. PLoS ONE 6: e22479. 21829627 10.1371/journal.pone.0022479 PMC3150374

[15] Cavagna A, Cimarelli A, Giardina I, Parisi G, Santagati R, et al (2010) Scale-free correlations in starling ocks. PNAS 107: 11865–11870. 20547832 10.1073/pnas.1005766107 PMC2900681

[16] Stöcker S (1999) Models for tuna school formation. Mathematical Biosciences 156: 167–190. 10204392 10.1016/s0025-5564(98)10065-2

[17] Liao JC, Beal DN, Lauder GV, Triantafyllou MS (2003) Fish exploiting vortices decrease muscle activity. Science 302: 1566–1569. 14645849 10.1126/science.1088295

[18] Dietrich G, Bredin J, Kerlrzin Y (2010) Interpersonal distance modeling during fighting activities. Motor Control 14 4: 509–527. 21051791 10.1123/mcj.14.4.509

[19] Boashash B (1992) Estimating and interpreting the instantaneous frequency of signal- part 1: fundamentals. Proceedings of the IEEE 80: 520–538.

[20] Boashash B (1992) Estimating and interpreting the instantaneous frequency of signal- part 2: algorithms and applications. Proceedings of the IEEE 80: 540–568.

[21] Pikovski A, Rosenblum M, Kurths J (2001) Synchronization: A universal concept in nonlinear sciences. Cambridge, MA: Cambridge University Press.

[22] Karvonen MJ, Kental E, Mustala O (1957) The effects of on heart rate a longitudinal study. Ann Med Exp Fenn 35: 307–315. 13470504

[23] Swain DP, Leutholtz BC (2007) Exercise prescription: a case study approach to the ACSM guidelines. Champaign, IL: THuman Kinetics Pub, 2nd edition.

[24] Taguchi M, Liao JC (2011) Rainbow trout consume less oxygen in turbulence: the energetics of swimming behaviors at different speeds. Journal of Experimental Biology 214: 1428–1436. 21490251 10.1242/jeb.052027 PMC3076074

[25] Schmidt RC, Carello C, Turvey MT (1990) Phase transitions and critical uctuations in the visual coordination of rhythmic movements between people. Journal of Experimental Psychology: Human Perception and Performance 16: 227–247. 2142196 10.1037//0096-1523.16.2.227

[26] Haken H, Kelso JAS, Bunz H (1985) A theoretical model of phase transitions in human hand movements. Biological Cybernetics 51: 347–356. 3978150 10.1007/BF00336922

[27] Kelso JAS (1995) Dynamic patterns: The self-organization of brain and behavior. Cambridge, MA: The MIT Press.

[28] Schmidt R, Fitzpatrick P, Caron R, Mergeche J (2011) Understanding social motor coordination. Human Movement Science 30: 834–845. 20817320 10.1016/j.humov.2010.05.014

[29] Nessler JA, Gilliland SJ (2009) Interpersonal synchronization during side by side treadmill walking is inuenced by leg length differential and altered sensory feedback. Human Movement Science 28 6: 772–785. 19796834 10.1016/j.humov.2009.04.007

[30] Riley MA, Richardson MJ, Shockley K, Ramenzoni VC (2011) Interpersonal synergies. Frontiers in Psychology 2: 1–7. 21716606 10.3389/fpsyg.2011.00038 PMC3110940

[31] Ramenzoni VC, Davis TJ, Riley MA, Shockley K, Baker AA (2011) Joint action in a cooperative precision task: nested processes of intrapersonal and interpersonal coordination. Experimental Brain Research 211: 447–457. 21479660 10.1007/s00221-011-2653-8

[32] Pecenka N, Keller PE (2011) The role of temporal prediction abilities in interpersonal sensorimotor synchronization. Experimental Brain Research 211: 505–515. 21424257 10.1007/s00221-011-2616-0

[33] Yokoyama K, Yamamoto Y (2011) Three people can synchronize as coupled oscillators during sports activities. PLoS Comp Biol 7: e1002181. 10.1371/journal.pcbi.1002181 PMC3188505 21998570

[34] Oullier O, de Guzman GC, Jantzen KJ, Lagarde J, Kelso JAS (2008) Social coordiantion dynamics: measuring human bonding. Social Neuroscience 3: 178–192. 18552971 10.1080/17470910701563392 PMC2156197

