# Supplementary material for: Switching Dynamics in an Interpersonal Competition Brings about “Deadlock” Synchronization of Players
Source: PLoS One. 2012 Nov 5;7(11):e47911. doi: 10.1371/journal.pone.0047911 (PMC3489899; doi:10.1371/journal.pone.0047911)
Supplement: Text S1 — Comment about zero sum nature of the sport game. (PDF) [file pone.0047911.s006.pdf]

Text S1: Comment about zero sum nature of the sport game.

it is important to note that scoring systems are different between games and may not reflect the absolute value of the game result for each team. For example, a three point win (1 for tie, zero for loss) scoring system would increase the goal of winning but not to tie because the difference between win-tie values (=2) is twice as great than tie-loss difference (=1). Such bias can arbitrarily control game behavior. It can increase a fun for spectator; however, in principle, each value for win and loss must be the same in absolute terms.
