# Supplementary material for: Switching Dynamics in an Interpersonal Competition Brings about “Deadlock” Synchronization of Players
Source: PLoS One. 2012 Nov 5;7(11):e47911. doi: 10.1371/journal.pone.0047911 (PMC3489899; doi:10.1371/journal.pone.0047911)
Supplement: Text S2 — Comment about the potential effect of inter-game interview on game behavior. (PDF) [file pone.0047911.s007.pdf]

Text S2: Comment about the potential effect of inter-game interview on game behavior.

Dawes, McTavish, and Shaklee [1] suggested that face-to-face discussion before dilemma decision task enhances the prediction about the other's strategy (i.e., the prediction about whether the other would defect or not). In the current experiment, the two players of a pair were interviewed together, so that one might suppose that this interview might have had an influence on the patterning of anti-phase synchronization. Certainly, each player would have found out what his opponent had been aware in previous trials. This information could contribute to form some prediction of their opponent's behavior. However, as described in text, contents of participant's report changed in parallel to anti-phase synchronization process. Thus, although we cannot know the extent this prediction affected game behavior, actual strategy exerted in the game would basically depend to opponent's step.

References

1. Dawes RM, McTavish J, Shaklee H (1977) Behavior, communication, and assumptions about other people's behavior in commons dilemma situation. J pers soc psychol 35: 1-11.
