# Supplementary material for: Switching Dynamics in an Interpersonal Competition Brings about “Deadlock” Synchronization of Players
Source: PLoS One. 2012 Nov 5;7(11):e47911. doi: 10.1371/journal.pone.0047911 (PMC3489899; doi:10.1371/journal.pone.0047911)
Supplement: Text S3 — Segmentation procedure according to two players' distance (). (PDF) [file pone.0047911.s008.pdf]

Text S3: Segmentation procedure according to two players' distance ($D(t)$).

Concept of the preparation and coordination segments were prescribed using a model of a Kendo match [1]. In the model, "preparation" was defined as the phase in which both players move on a oscillatory fashion (and consequently, the distance between them also oscillates). In the current study, we determined the time point of boundary between two segments (t_b) as initial point at which linear regression coefficient of $D(t_b : t_b - 15 \leq t_b < t_b + 15)$ turned to positive.

References

1. Dietrich G, Bredin J, Kerlrzin Y (2010) Interpersonal distance modeling during fighting activities. Motor Control 14(4): 509-527.
