# Supplementary material for: Switching Dynamics in an Interpersonal Competition Brings about “Deadlock” Synchronization of Players
Source: PLoS One. 2012 Nov 5;7(11):e47911. doi: 10.1371/journal.pone.0047911 (PMC3489899; doi:10.1371/journal.pone.0047911)
Supplement: Text S4 — Effectiveness of Hilbert transform on current data. (PDF) [file pone.0047911.s009.pdf]

Text S4: Effectiveness of Hilbert transform on current $SV(t)$ data.

Hilbert transform is suit to extract phase information from relatively slowly varying one dimensional time-series signal such as $SV(t)$ in our data. It is difficult to detect characteristic time events (e.g. a time of peak in a cycle) so that phase information would be difficult to extract from them. Hilbert transform procedure is one of the solutions for this problem in that it transforms a one dimensional scalar signal ($s(t)$) into complex form ($s(t) + is_H(t)$), and calculates the phase for every point of $s(t)$ as an angle on ($s(t)$, $s_H(t)$) plane. This calculation is effective to calculate phase for all the points within a characteristic period and obtain sufficient statistics for the determination of the phase relationship. We subtracted the mean value from original $SV(t)$ signal before transformation as the calculated value is sensitive to low-frequency trends [1].

References

1. Pikovski A, Rosenblum M, Kurths J (2001) Synchronization: A universal concept in nonlinear sciences. Cambridge, MA: Cambridge University Press.
